# The Landscape of Movement Control in Locomotion: Cost, Strategy, and Solution

**DOI:** 10.3389/fpsyg.2019.00716

**Published:** 2019-04-09

**Authors:** James L. Croft, Ryan T. Schroeder, John E. A. Bertram

**Affiliations:** ^1^Centre of Exercise and Sports Science Research, School of Medical and Health Sciences, Edith Cowan University, Joondalup, WA, Australia; ^2^Biomedical Engineering, University of Calgary, Calgary, AB, Canada; ^3^Cumming School of Medicine, University of Calgary, Calgary, AB, Canada

**Keywords:** cost surface, energy, energetic cost, gait, constraints

## Abstract

Features of gait are determined at multiple levels, from the selection of the gait itself (e.g., walk or run) through the specific parameters utilized (stride length, frequency, etc.) to the pattern of muscular excitation. The ultimate choices are determined neurally, but what is involved with deciding on the appropriate strategy? Human locomotion appears stereotyped not so much because the pattern is predetermined, but because these movement patterns are good solutions for providing movement utilizing the machinery available to the individual (the legs and their requisite components). Under different circumstances the appropriate solution may differ broadly (different gait) or subtly (different parameters). Interpretation of the neural decision making process would benefit from understanding the influences that are utilized in the selection of the appropriate solution in any set of circumstances, including normal conditions. In this review we survey an array of studies that point to energetic cost as a key input to the gait coordination system, and not just an outcome of the gait pattern implemented. We then use that information to rigorously define the construct proposed by Sparrow and Newell (1998) where the effects of environment, organism, and task act as constraints determining the solution set available, and the coordination pattern is then implemented under pressure for energetic economy. The fit between the environment and the organism define affordances that can be actualized. We rely on a novel conceptualization of task that recognizes that the task goal needs to be separated from the mechanisms that achieve it so that the selection of a particular implementation strategy can be exposed and understood. This reformulation of the Sparrow and Newell construct is then linked to the proposed pressure for economy by considering it as an optimization problem, where the most readily selected gait strategy will be the one that achieves the task goal at (or near) the energetic minimum.

## Introduction

**FIGURE 1:**
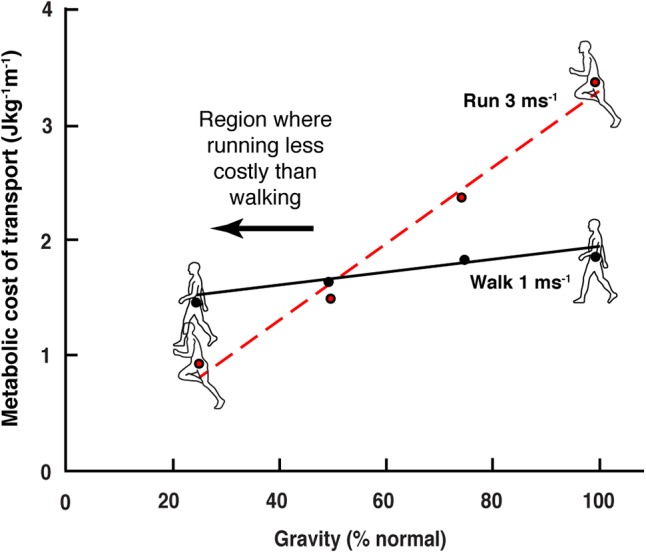
Reduction on the cost of walking and running in simulated gravity (data extracted from Farley and McMahon, 1992). In normal earth gravity the energetic cost of running (per distance) is greater than walking. As gravity level is reduced, the energetic cost of running decreases more than that of walking. At approximately 50% normal gravity, the energetic cost of the two gaits is equivalent, and below this level walking cost is greater than that of running.

When an individual walks into the water at a beach he/she will usually switch to a bounce-like running gait as they reach chest depth. Unlike on dry land, the gait change happens even though the individual travels quite slowly. At chest depth the buoyancy of the body roughly simulates a reduction in gravity of slightly more than 50%. It has long been known that the energetic cost of running decreases directly as gravity is reduced, while the cost of walking is far less sensitive to this effect (Farley and McMahon, 1992; Donelan and Kram, 1997; Kram et al., 1997; Bertram and Hasaneini, 2013). The differential decrease in locomotion cost means that at approximately 50% Earth’s gravity walking and running costs are equivalent, and at lower gravity the cost of running is less than that of walking (Figure 1).

The spontaneous transition from walking to running in chest deep water, where the energetic cost of the run stride is slightly less than the walking stride, suggests that the human body can be remarkably sensitive to the energetic opportunities available in its environment. It appears that this can have a direct effect on the motor control strategy implemented, such as stimulating a change in gait from a walk to a run. We have recently demonstrated that this sensitivity to the energetic opportunities afforded by the unusual circumstances of simulated reduced gravity can involve the spontaneous implementation of quite subtle and non-intuitive movement strategies (such as making low gravity running less bouncy, Polet et al., 2018), indicating that energetic savings, particularly in locomotion, may be highly valued by the motor control system. This also indicates that substantial plasticity is available to the control regime even for an activity as seemingly stereotyped as locomotion.

It has long been recognized that gait selection is likely based on the energetic effectiveness of the particular movement pattern, so each gait is generally selected over the limited speed range in which its energetic cost is less than alternative gaits (Hoyt and Taylor, 1981; Alexander, 2002). Gaining proficiency at arm control in a reaching task, while maintaining a specific level of speed and accuracy, also results in reductions in metabolic cost (Huang et al., 2012), suggesting that motor control adjustments in general may in some way be linked to a cost assessment of the control strategy options available. Still, energy minimization in arm movements remains a contentious issue due to conflicting evidence (Kistemaker et al., 2010) and the success of alternative optimization goals in predicting movement patterns of the upper limbs (e.g., jerk; Hogan, 1984; Kistemaker et al., 2014).

Ultimately it would be useful to identify and understand the mechanism(s) used to make such assessments, and their sensitivity and limitations. At this point, however, there is substantial ambiguity regarding even the circumstances in which energetic influences can be resolved (Wong and Donelan, 2017). Fully understanding the ‘priority landscape’ that the central nervous system (CNS) operates within remains an important aspect of understanding priority recalibration in movement strategy selection, where prioritization could involve varying the weighting of various options such as force, force rate, acceleration, jerk, etc. or the energetic consequences of these and other functional characteristics. Fully recognizing and understanding the mechanisms involved could provide novel opportunities to influence gait control. More obvious and direct interventions might be replaced by potentially subtle effects that influence aspects of the energetic cost landscape and CNS sensitivity to it. Recognizing the interaction between cost and control can also provide a foundation for reinterpreting neural control models.

**FIGURE 2:**
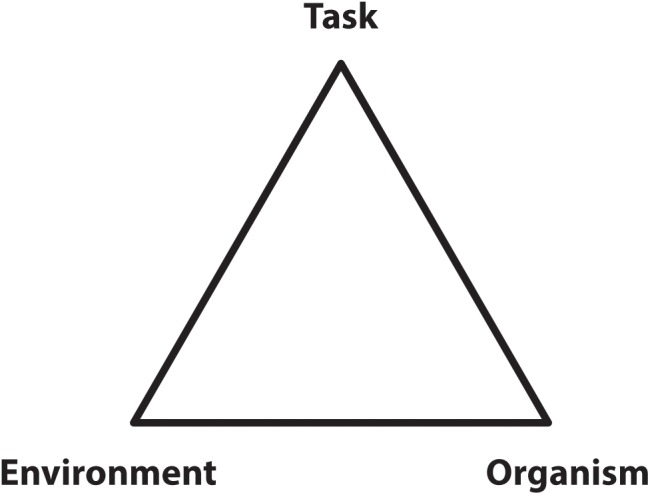
A diagram of the three types of constraints influencing locomotion control as conceptualized by Sparrow and Newell (1998). The image is meant to indicate the bounds applied by each of these influences so that locomotion, then, would be constrained to occur within these bounds and guided by pressure to operate economically.

Sparrow (1983) and Sparrow and Newell (1998) proposed a role for energetic economy in the formulation of movement strategies and the process of learning new tasks. Their 1998 paper partially formalized this general perspective by recognizing that organismic, environmental, and task dependent factors can act as constraints on the selected movement strategy (Figure 2) while suggesting the selection of specific control patterns would be guided by a ‘pressure to operate economically.’

The objective of the current contribution is twofold. The first is to review literature that investigates the range, form and apparent sensitivity of gait to energetic cost. The intention is to focus critical attention on this aspect of motor control that is sometimes overlooked and, more importantly, is not yet fully understood. The second is to build on this to formulate a more rigorous definition of the Sparrow and Newell construct in order to provide a context in which further quantitative experimental investigation on the topic can proceed.

## Cost: Another Surface We Move On

**FIGURE 3:**
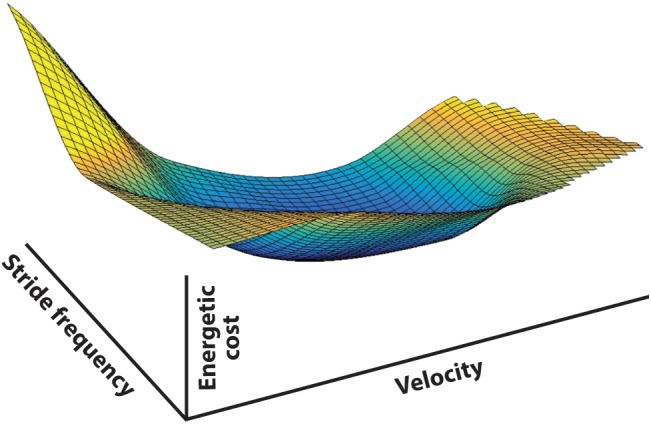
The energetic cost surface of human walking represented in stride frequency, translational velocity, and metabolic cost space. The surface is derived from 10 subjects walking at prescribed treadmill speeds while pacing their steps to a metronome – consequently the gait parameters were fully prescribed and covered the full range of physically possible walking frequency-speed combinations (data from Bertram, 2005). The metabolic energy cost surface represents the cost associated with level walking using any combination of gait parameters, at least with symmetric steps. Any walking strategy selected by the central nervous system will be represented by a point on this surface, demonstrating that the choice of any given set of gait parameters is associated with an energetic cost to the system. Iso-cost contours are shown in 2D in Figure 4.

In walking, speed (*v*), stride length (*d*_s_), and stride frequency (*f*_s_) are irrevocably linked (*v* = *d*_s_ × *f*_s_). Meanwhile, there is a mechanical, and consequently a metabolic, cost for any combination of *v*, *d*_s_, and *f*_s_. There is no ‘free lunch’ in legged locomotion, and so any set of movements comes with a definable cost. Combinations of *v*, *d*_s_, and *f*_s_ determine all ways to walk (at least with symmetric steps). Since each combination comes with a specific metabolic cost, the full range of possible gait parameter combinations produces a ‘cost surface’ in speed-frequency-metabolic cost space (Figure 3). For healthy human walking this surface has the general shape of a ‘bowl’ with a global minimum. When no parameters are externally determined the individual is free to select any *v*, *d*_s_, *f*_s_ combination, but this usually results in arriving at or near the global energetic cost minimum, the unique combination that requires the least metabolic investment (preferred walking speed with normal self-selected frequency and step length). Does the individual naturally ‘prefer’ this set of gait parameters because of some pattern determined by the CNS or is cost a guiding input that directs the choice to provide this as the best result? Although fully recognizing that numerous factors influence the final gait expression, based on the evidence described in the following, we suspect it is no accident that spontaneously preferred and energetically cost-effective walking speeds tend to coincide, as they appear to in animal locomotion as well (Hoyt and Taylor, 1981).

### Constrained Optimization: Energetic Influence on the Plasticity of Motor Control

Increasing above preferred walking speed involves using a combination of altered *d*_s_ and *f*_s_ that keeps the subject at a local cost minimum with respect to speed (Kuo, 2001a). Greater speed displaces the cost from the global minimum, but subjects then choose gait parameters (*f*_s_ and *d*_s_) with the minimum cost available for the imposed speed (they move along the ‘valley bottom’ of the cost surface stretching from the global minimum toward the limit of walking speed). Maintaining this local minimum with respect to speed is what we consider the ‘normal’ speed-frequency relationship. Speed adaptation appears to be accomplished such that the speed is achieved with the least metabolic investment by selecting the gait parameters that satisfy the task goal with the least energetic cost (Kuo, 2001b). It is certainly possible to walk with a different set of gait parameters at both high and low speeds, however, alternate solutions are not selected since they have a greater energetic cost of transport than could otherwise be achieved (i.e., they are energetically non-optimal). The control required to realize this condition in normal circumstances could derive from evolutionary adaptation of the species in general, where the motor control pattern is determined through natural selection at the species level (those ancestors that employed a more costly movement regime were at a survival disadvantage, so their genes were eventually removed from the gene pool). More likely, however, it is derived from local and immediate assessment of the energetic optimum strategy (based on the adaptive plasticity observed in the studies following).

Like the normal speed-frequency relationship, most features of walking and running appear stereotyped. This is one of the most compelling of Bernstein’s conclusions (Latash et al., 1996): that there are a given set of activity patterns characterizing the fundamentals of locomotion. However, it is becoming more and more evident that the apparent stereotyped movement patterns are not stereotyped at all, but are simply a common, solution that is routinely selected as a result of its energetic advantage. It is relatively easy to demonstrate that various aspects of the apparently pre-determined patterns of locomotion are sensitive to a variety of influences (Nessler and Gilliland, 2009; Richardson et al., 2009).

**FIGURE 4:**
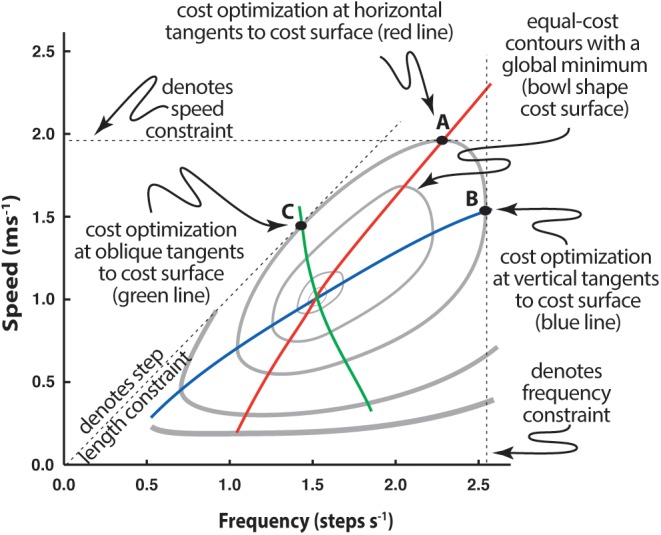
Determination of speed-frequency-step length selection when a constraint is applied to walking. Individuals walking in registry to a range of metronome beats exhibit a different speed-frequency relationship (blue line) than those walking over a range of prescribed speeds (as on a treadmill; red line). Likewise, a third speed-frequency relationship is observed if step length is prescribed by stepping on floor markers (green line). Speed-frequency relations are presented with respect to the metabolic cost surface, here represented by cost contours (gray lines) that describe the general ‘bowl’ shape of the surface, where the lowest cost occurs within the innermost contour and each contour indicates a cost greater than the one internal to it. See text for further explanation.

Simple experiments can demonstrate that energetic cost has a surprisingly fundamental influence on relatively subtle adaptations. For instance, the speed-stride frequency relationship when walking on a treadmill, generally regarded as normal, is markedly different from that used when the same individual walks along a hallway with step frequency guided by a metronome, or when pace lengths are guided by floor markers (Bertram and Ruina, 2001; Bertram, 2005). In each case the task of walking is affected by a functional constraint: constrained *v* on a treadmill, constrained *f*_s_ with a metronome, and constrained *d*_s_ with floor markers (Figure 4).

Constraining the strategic options of locomotion to two interdependent parameters provides an interesting opportunity to investigate the process through which the CNS selects the solution to the problem of producing effective locomotion. CNS control is particularly interesting for constrained *f*_s_ or *d*_s_ because these are not conditioned behaviors and are likely far less influenced by long-term experience (it is likely that the selection of speed is a natural operative in walking so experimental manipulation of this parameter simply results in a control pattern we recognize as normal and provides little information on how that pattern is arrived at). For either constrained *f*_s_ or *d*_s_, however, speed is left free to be adjusted, as well as the remaining unconstrained gait parameter (*d*_s_ for *f*_s_ constraint, *f*_s_ for *d*_s_ constraint). The individual selects speed and the free parameter that emerges at a cost minimum along the track of that constraint (Figure 4). The resulting speed-frequency relationship differs from normal and one might assume this is because the constraints interfere with normal gait. However, each feels natural to the subject, likely because he/she is moving in the most appropriate way given the circumstances (explained in the following).

Gait parameter adjustment appears to result from the constraint affecting the energetic ‘cost optimization’ of the task (Bertram and Ruina, 2001; Bertram, 2005). Since *v*, *d*_s_, and *f*_s_ are so intimately related, constraining any one of the three means that any change in one of the two remaining parameters must be compensated for by the other. In Figure 4 the effect of a constraint can be seen by following the dashed line associated with its axis. For instance, for speed constrained walking (as on a treadmill) the subject must maintain the set speed. This is represented by the horizontal dashed line. It is physically possible to use a broad range of step frequencies to walk at this speed, but the frequency at which that speed can be maintained for the least energetic cost will occur when the horizontal line is tangent to the cost surface (cost contour on a planar plot), as indicated by point A. The energetically optimal speed-frequency relationship for speed constrained walking, then, will be a series of horizontal tangents to the contours (red line). Likewise, a frequency constraint (overground walking to a metronome beat) is a vertical slice through the cost surface (vertical dashed line, Figure 4). Under this constraint, the most energetically effective speeds associated with prescribed frequencies are found on the respective vertical line and tangential to the cost contour (point B). Thus, the optimum speed-frequency relationship for frequency constrained walking is a series of vertical tangents to the surface (blue line).

Step length is not directly indicated as an axis on this plot, but it is implied due to its relationship to speed and frequency (where *v* = *d*_s_ × *f*_s_). From this relationship step length appears as a slope on a plot of speed vs. frequency (note for instance that a line emanating from the origin with a steep slope means that speed increases substantially for a small increase in frequency – as would be the case for long step lengths – whereas a shallow slope would indicate that speed increases marginally for the same increase in frequency, as expected for short step lengths). For prescribed step lengths, the most energetically cost effective speed and frequency occur when the slope representing the step length is tangent to the cost surface (point C, Figure 4) and the speed-frequency relationship optimum for step length constrained walking is a series of tangents to slopes emanating from the origin (green line).

The similarity between cost surface optimization and the selection of gait parameters by subjects (Bertram and Ruina, 2001; Bertram, 2005) leads us to conclude the CNS preferentially selects gait parameters that minimize energetic cost under imposed constraints. That is, the energetic cost profile influences the movement strategy implemented by the CNS, where the strategy will involve features down to the specific parameters utilized. We have shown that this also holds for running (Gutmann et al., 2006), hopping (Gutmann and Bertram, 2013), and walking in cats (Bertram et al., 2014).

### Dynamic Manipulation of the Cost Surface

The example above is compelling, but involves only simple manipulations of options available on an existing cost surface. Consequently, the solution is a relatively modest variation from normal. More direct evidence would demonstrate that the solution can be modulated in dynamic response to changing optima or manipulations of the cost surface itself.

Snaterse et al. (2011) altered the relationship between *f*_s_ and *v* by measuring *f*_s_ and processing it through a computer algorithm to dynamically control treadmill speed. From this, and knowing the shape of the energetic cost surface, they were able to show that individuals spontaneously responded to the algorithm to use an option that was (unconsciously) perceived to be less energetically demanding. This was the case if the less costly option involved slowing down, speeding up, or oscillating between the two (depending where on the energetic cost surface the individual was and the artificial speed-frequency relationship imposed).

Results of that study also suggested that gait accommodation occurred in two distinct stages, one rapid – on the order of 1–2 s, and one slow – on the order of several tens of seconds. They hypothesized that the two-part response was driven by different mechanisms: the rapid response, likely based on feed-forward internal estimation of movement options and the slower response likely resulting from proprioception and physiological feedback that allowed ‘tuning’ of the initial guess. In both cases, however, a key factor determining the motor control solution appropriate under the imposed conditions appears to be energetic cost.

In another study the cost surface was directly manipulated using a small computer-controlled exoskeleton that applied dynamic resistive torques to the knee (Selinger et al., 2015). Through control of this device the energetics of walking were influenced by penalizing either lower or higher than preferred *f*_s_ (in independent experiments). Three particularly informative features were observed.

Individuals minimized energetic expenditure, even in such an artificially imposed circumstance. However, many individuals could not discover the altered minimum until forced to explore the novel cost surface by guiding them through different combinations of *f*_s_ and *d*_s_. When these same subjects were freed of the exploratory constraints and provided with the new artificially imposed cost energetics, subjects spontaneously located the new cost optimum. This finding held if the experiment was initiated with subjects constrained to a set of walking parameters (by having them match their gait to a metronome) at stride frequencies above *or* below the artificially determined optimum. Once released from the frequency constraint subjects shifted to a gait that had the least energetic cost (even though this required a different than normal speed-frequency relationship).

Interestingly, there was a latency in returning to normal walking parameters once the exoskeleton influence was turned off. Although appearing to be counter-evidence regarding the sensitivity of cost in controlling gait, this could also be an important clue regarding how energetic cost influences CNS control (Bertram, 2015). The imposed artificial cost surface involved a modest global increase above normal walking, but with the optimum shifted to a new stride frequency (higher or lower than normal). Thus, when the exoskeleton was turned off, the body likely perceived a meaningful decrease in energetic cost even without a gait adjustment. It appears the feed-forward component of the control was not motivated to seek an even greater decrease in cost (i.e., locating the global minimum on the less costly surface); the adjustment to the new surface appeared delayed due to the immediate positive pay-off for the movement strategy that was currently in use. Nevertheless, subjects eventually rediscovered their unconstrained preferred gait parameters.

Of particular importance was the study design in this case. The external torque device was controlled so that some of the competing neural control advantages could be eliminated. For instance, the artificially imposed energetic optima were purposely designed to be different from the knee torque minimum, making identification of energetic cost as the motivation for the gait optimization much more robust.

## The Scope of Plasticity

The studies above indicate that locomotion control has the capacity to change in response to different constraints and these appear related to energetic cost. How far do these adaptations reach?

### Split Belt Accommodation and Adaptation

One protocol useful for examining locomotion control strategies is split-belt walking, where a subject walks on a treadmill with independently controlled belt speeds for the left and right legs (Prokop et al., 1995; Jensen et al., 1998). Subjects can immediately accommodate different belt speeds but do so with a markedly asymmetric gait. After some period (5–10 min) the motor control system adapts to provide a smoother gait, often described as ‘symmetric’ (Malone et al., 2012). Of course, the impression of symmetry following adaptation to the differential speed of the belts can only be with regard to general body motion, as the legs must move and be controlled in an asymmetric manner to manage the asymmetric functional environment each leg experiences (Reisman et al., 2005; Selgrade et al., 2017). Although healthy individuals adapt to asymmetric split-belts in consistent ways, one might ask – why do they adapt at all? Even with an asymmetric gait these individuals are not in jeopardy of failing to maintain their position on the treadmill (it takes several minutes to fully adapt, after all). It is common to assume the ‘healthy nervous system favors a smooth, symmetric gait’ (Malone et al., 2012), but maintaining an appearance of a symmetric gait in this circumstance requires quite asymmetric limb kinematics, neuromuscular activity, and kinetics.

**FIGURE 5:**
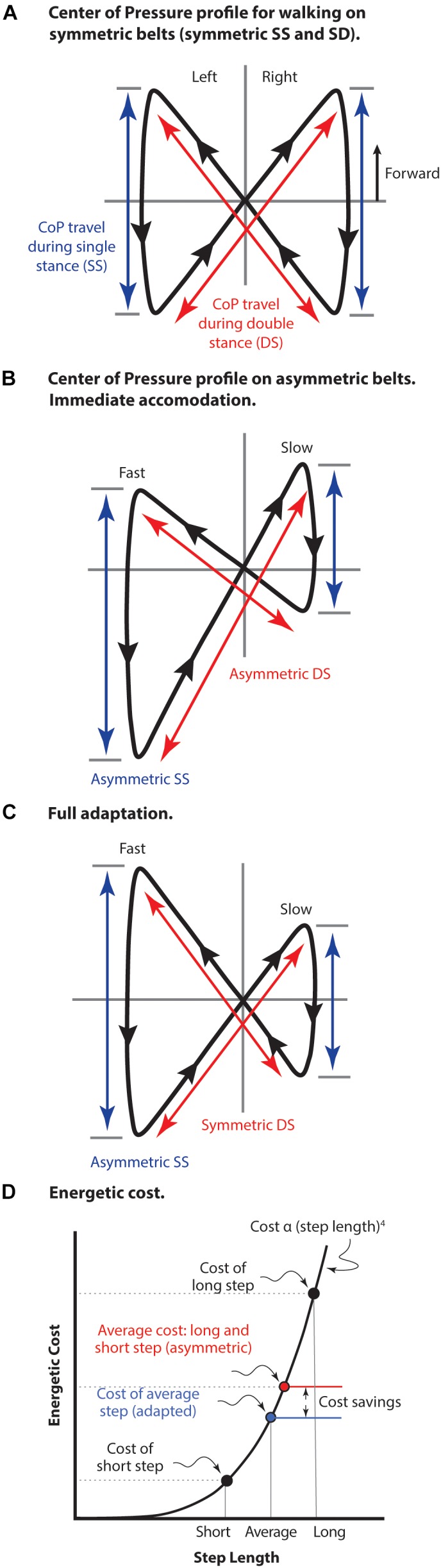
The center of pressure (CoP) profile for split belt treadmill walking. **(A)** Normal walking with both belts moving at the same speed. The travel of CoP during both single limb stance (SS) and during double stance (DS) is equal giving a truly symmetrical pattern. **(B)** Early adaptation immediately following shift in belt speeds to asymmetric (left fast and right slow). Position on the treadmill is maintained with both asymmetric single and double stance components of the stride. **(C)** Following adaptation to asymmetrical belt speeds. Travel on the fast belt remains farther than on the slow belt during SS, but the travel during DS becomes equivalent. As well, the foot position at beginning and end of SS becomes aligned relative to the DS cross-over point. Figure adapted with permission from Fujiki et al. (2015). Mawase et al. (2013) used a dual-rate motor control process to model these pattern adjustments. **(D)** The energetic cost of locomotion is represented as an arbitrary function of step length raised to the fourth power (not to scale). The average cost of a long step and a short step is compared to the cost of an average step length, as well as the resulting cost savings. This cost saving is due to the non-linearity of the relationship in which slope increases with step length. The strategy of taking an average step length is employed for both symmetric belt walking as well as asymmetric belt walking after full adaptation has occurred.

The center of pressure (CoP) profile conveniently illustrates both symmetric and asymmetric features of split-belt treadmill gait (Figure 5). The fully symmetric CoP pattern of normal walking occurs when belt motion is equal (Figure 5A). In this case the movement during both the single limb stance portion of the stride and the double stance portion (step-to-step transition) are equivalent for both limbs and single stance travel is centered around the position that the double stance trajectories intersect. Immediately following a shift to asymmetric belt speeds (Figure 5B) position on the treadmill is maintained using a gait with unequal portions of both single stance and double stance for each leg. As well, the single stance begins and ends in different positions on each belt making the cross-over point of double stance different relative to each single limb stance. Following adaptation (Figure 5C) double stance travel becomes equivalent even while single limb stance travel necessarily remains asymmetric. Like symmetric belt walking, single stance travel in both limbs becomes centered around the double stance cross-over point.

Mawase et al. (2013) propose that these pattern changes are consistent with a dual-rate control process, reminiscent of Snaterse et al. (2011), where feedback and feed-forward processes are involved. They suggest motor control actions during single limb stance are driven by a feedback loop monitoring environmental conditions. As a result, subjects exhibit a rapid response to changes in belt speeds. They remain in position by generating different single limb stance position changes, however, double stance adaptation takes more time (on the order of minutes). This likely indicates true adaptive learning that makes the gait as symmetrical as possible under the circumstances. True adaptive learning in a cyclical behavior is typically marked by post-adaptation relearning and Mawase et al. (2013) point out that a relearning phase is only found in the slow rate response of double stance. Regardless of the roles for feedback and feed-forward processes in split belt walking, it is still reasonable to ask why longer-term adaptation occurs at all. The accommodation immediately following a shift to asymmetric belt speeds already allows the individual to maintain position on the treadmill. What motivation does the motor control system have for further adaptation?

There is one practical advantage – that of reduced energetic cost. Note that other candidate issues such as muscle force or joint torques can be eliminated simply because symmetric walking (with symmetric leg function) at either the slow or fast belt speed is not problematic. The issue being dealt with is at the whole body level. Direct evidence of the energetic advantage of adaptation is provided by Finley et al. (2013) who demonstrated a decreased metabolic cost after full adaptation to asymmetric split-belt walking.

How could energy be ‘saved’ by adaptation? Positive and negative work are performed at various times during the gait cycle (Donelan et al., 2002; Kuo et al., 2005) but a large portion of energetic cost for walking occurs at the transition from one stance leg to the other (Kuo, 2001b; Bertram, 2015). A variety of strategies are employed to minimize this cost during normal walking, such as choice of step length and the critical timing of previous stance leg push-off relative to heel-strike of the next stance limb (Donelan et al., 2002; Kuo et al., 2005). This critical timing does not simply add forward acceleration, but also adjusts the orientation of the center of mass (CoM) velocity vector in a manner that reduces step transition costs – a portion of the gait cycle where substantial energetic cost originates (Kuo, 2001b; Donelan et al., 2002). In contrast, little energy needs to be added during the single stance phase of walking, which relies on passive inverted pendulum-like conversion between kinetic and potential energy (Srinivasan and Ruina, 2006).

The cost of the step-to-step transition (double stance) is proportional to step length raised to the fourth power (Kuo, 2001a). A consequence of this non-linear relationship is that alternating step lengths results in a higher average energetic cost compared to an average step length in both legs (Figure 5D). Walking energetics tend to rely on an optimal compromise between energy losses and the cost of the mechanisms used to replace those losses (Srinivasan and Ruina, 2006; Bertram and Hasaneini, 2013), so the specific strategy utilized to deal with the challenges of asymmetric split belt walking will depend on a combination of (yet to be determined) factors. However, it appears clear that the motivation for adaptation to asymmetric split belt walking resides with reduction of energetic cost, rather than any issue of CNS ‘preferred’ symmetrical activity (unless the interpretation is that the CNS prefers a lower energetic cost option, if available). Given that some of the adaptations explicitly influence whole-body dynamics and not just limb positioning, it is difficult to imagine how such control could be expressed without a cost-related input. For instance, ankle push-off could occur slightly later in double stance and still compensate for rearward translation during the fast belt step. However, the critical timing of these events is specifically tuned to minimize muscle work required to resupply energy lost from the system during the step. Likewise, it is possible to compensate with added work at the hip, but this is less cost-effective than the critically timed ankle push-off (Kuo, 2001b). Without a functioning ankle, unilateral *trans*-tibial amputees are forced to use the hip torque strategy (Selgrade et al., 2017), demonstrating it is a viable strategy for maintaining position on the asymmetric belt, but it is not an option selected when a more cost-effective strategy is available (to non-amputees).

### Simulated Gravity Responses

Gravity has a ubiquitous physical influence on normal locomotion, so it can be assumed that walking and running are fully adapted to contend with the effects of native gravity. Using a harness with near constant upward force can simulate the effects of a reduced gravitational acceleration on the CoM. As discussed in the Section “Introduction,” simulated reduction in gravity has a remarkably modest effect on the cost of walking whereas running costs are reduced directly and substantially with a decrease in gravity (Farley and McMahon, 1992; Griffin et al., 1999; Donelan and Kram, 2000), so that at sufficiently low gravity levels (below 40–50% Earth’s gravity) the cost of running is less than that of walking. This may provide an explanation for why astronauts are seen using running and skipping gaits in videos from the lunar landings (Moon gravity is 17% that of Earth). Such behavior also indicates that astronauts recognized walking as a less efficient gait in an extremely unusual circumstance; they responded by spontaneously altering coordination patterns and adapting to the energetic opportunities of a foreign gravitational environment (just as beachgoers do). The astronauts did not actually ‘walk’ on the moon, likely because their CNS perceived walking as a less economical gait in this unusual environment.

It must be acknowledged that the dramatic change in gravity could make the well-conditioned force generating strategy of the legs quite inappropriate (as they are set for normal gravity). However, numerous studies indicate that muscle gain settings are fairly rapidly adjusted to artificially altered gravity effects. The question being addressed here is, what information is being utilized to determine the appropriate gain setting for the new operational conditions. Recent simulated reduced gravity studies indicate an impressive adaptive control regime appears to be at work. Hasaneini et al. (2013) used a dynamic self-optimizing model (slightly more complex than Srinivasan and Ruina, 2006) and compared it with human subjects in simulated reduced gravity. The model predicts subtle changes in step length with gravity, and this was confirmed in human subjects. The model can be decomposed to determine the reason step length changes are optimum in reduced gravity. For running the energetic losses per foot contact remain approximately the same as on earth, but the non-contact flight time will be longer in reduced gravity. This means that for each contact (energy loss) much more distance is covered, resulting in a direct decrease in energetic cost as gravity is reduced. The effect is more subtle in reduced gravity walking, where the body mass does not need to slow down (as much as in higher gravities) as it vaults over the leg during single stance. As a result, it does not need to travel as fast during the rest of the stride to maintain the same average forward speed. Longer step lengths are allowed because the slower motion at contact means less energy is lost to the step-to-step transition at a slightly longer step length (and slower speed). The subtle nature of this advantage strongly suggests that the energetic consequences of locomotory movements are not simply monitored, but have a strong influence on how the CNS controls the detailed function of the limbs in locomotion.

### Energetic Cost Influence on Gait Selection Strategy

The evidence above suggests that metabolic cost has an effect on both the selection and tuning of movement parameters; cost is not simply an outcome of those selected parameters. A striking example of the complex influence of energetic cost is seen in the results of a clever study showing that metabolic cost is implicated in what might be considered higher-level features of gait, the selection and partitioning of different gait modes, in addition to how the limbs are used within a gait (Long and Srinivasan, 2013). This was also suggested in both the gait change observed when wading into water at the beach and in the astronauts on the moon, selecting a run-like gait at slower than normal speeds because reduced (effective) gravity meant the run had lower transport costs than walking.

Long and Srinivasan (2013) instructed subjects to move from a starting point to a defined destination (on the order of 120 m with the end-point visible) in a prescribed time interval. Subjects were provided with a countdown timer to monitor progress and were instructed to arrive at the end point precisely as the timer counted down to zero. The time interval varied from one that was too short to reach without running the entire distance to one that was so long the subject would reach the end too quickly if they walked at a natural pace. For intervals that required subjects to walk slower than preferred they chose a combination of walking and resting periods; for intervals requiring walking faster than preferred but slower than preferred running speed, subjects chose a combination of walking and running. Although they switched back and forth between gaits for intermediate time intervals, subjects traveled at the speed that was most economical within that gait. Interestingly, regardless of the target time, subjects spontaneously combined gaits in such a way that allowed them to traverse the distance near the most economical strategy available. This result demonstrates some nuances that warrant a more detailed description.

**FIGURE 6:**
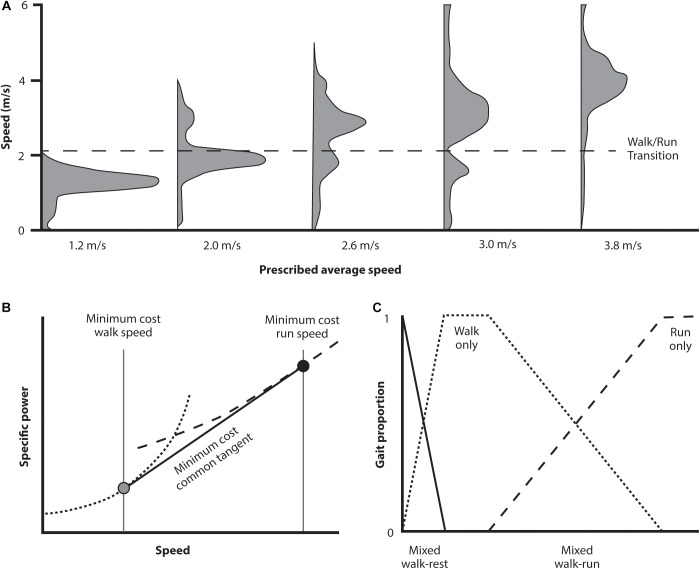
Abridged results from a study investigating spontaneously selected movement strategy when subjects are given a distance to traverse in a prescribed time period (where subjects are aware of both the end goal and the time elapsed). In **(A)** (above) the distribution of speeds selected for a selected group of traverse times is indicated. For traverse times at each extreme, that can be managed at a walk or demand a run, then the predominant gait is a walk or run at the preferred speed. For intermediate traverse times, however, the subjects select a combination of walk and run gaits in the proportion needed to achieve the distance in the prescribed time. Of interest is what calculation is needed to determine the appropriate speeds to accomplish this task with the least energetic cost **(B)**. The specific power curves for these two gaits are concave upward. For traverse times intermediate between the preferred walking and running speeds the most economical strategy is to select a combination of gaits using the speed for each of the tangent line between (gray and black circles). Any traverse time can be economically accomplished using a combination of these two speeds depending on the proportion of each gait selected **(C)**.

The key results of the Long and Srinivasan study are shown in Figure 6. The subjects’ behavior over the prescribed intervals is shown in panel A. As the prescribed time interval decreases below that which can be traversed at preferred walking speed, subjects combined periods of both walking and running. Rather than selecting a gradient of walking and running speeds, however, they selected a discrete economical walking and running speed and shifted between the two optima to traverse the distance at an appropriate average pace. Over the range of time intervals, the proportion of walking and running gaits shifted in a systematic way that again resulted in the most economical traverse of the distance in the interval allotted, a result that requires a very sophisticated calculation of the energetic expenditures involved.

Long and Srinivasan (2013) noted that walk and run power curves are convex upward. For such curves the common tangent indicates the minimum composite travel cost (Figure 6B). Since energetic cost of walking or running over the range of this tangent are greater than at the tangent itself, any intermediate pace can most economically be generated by combining the appropriate proportion of walking and running at the speeds where the tangent contacts each gait’s cost curve (Figure 6C). It is this proportional combination that explains the speed and gait distribution empirically (and spontaneously) selected (Figure 6A). This indicates these individuals possessed an innate awareness of the energetic landscape (of walking, running, and the combined effects of both) and the CNS utilized this effectively. This was the case even though it involved combining gaits and speeds to generate an overall ‘most economical’ transit strategy under varying defined intervals. Participants accomplished this without specific practice at the task, nor any conscious understanding of precisely what they were doing – it was just what seemed natural (because they spontaneously selected this locomotion strategy from the host of alternatives).

## How Should Locomotion Be Controlled, and for What Purpose?

The studies described above each provide an important piece of evidence indicating that gait coordination strategy is likely heavily influenced by energetic cost. Due to the circumstantial nature of the evidence, however, it could be that energetic cost only appears to guide the movements, and the association with minimum cost results as a coincidence of some other unidentified influence. Although this remains a possibility, the range of circumstances where economy of motion is maintained, and the consistency of the accumulated results that each depend so directly on energetic cost, makes it difficult to conceive of another feature that could be responsible for this result simply as a matter of coincidence. We are left, then, to ask the question: what is (are) the mechanism(s) through which energetic economy is assessed and influences motor selection?

Locomotion is controlled by the CNS, however, the brain must operate the body within functional limits to move in effective ways. In this review we have gathered evidence indicating that energetic cost appears to be an important, if sometimes underappreciated, aspect of the roadmap that the brain follows to determine what an ‘effective’ movement strategy is under the imposed circumstances.

If the motor control system follows this roadmap, how does it get its directions? Snaterse et al. (2011) found compelling evidence for a two-part response system: one rapid, acting on the order of a few seconds (interpreted as feed-forward control positioning the system as well as possible to deal with immediate challenges, particularly as they change – which we have referred to as ‘accommodation’ in the context of immediate adjustment to asymmetric split belts), the other was slower, acting on the order of tens of seconds or more (interpreted as a feedback, ‘tuning’ the system to operate optimally within the new circumstances – what we have referred to as ‘adaptation’). A forward model anticipates the next state of the system based on its current state and estimates the actions required to fulfill the goal of the movement regime. Feed-forward control in circumstances not previously experienced, such as the dissociation of the speed-frequency relationship of the Snaterse et al. (2011) study, astronauts on the moon, or even simple frequency and step length constraints (Bertram, 2005), suggests an anticipation of the cost surface form, where a previous somewhat related experience may help to inform the consequences of implementing a particular movement strategy. This is an area where very little is currently understood, but one that holds substantial potential for insight into mechanisms involved with gait coordination. However, even initial experimentation in this area requires an appreciation and understanding of the cost surface the motor control system interacts with. Although energetics often arises as a suggested objective function that can guide parameter selection (Alexander, 1991; Kuo, 2001b; Sawicki and Ferris, 2009), no consensus to its role currently exists and numerous other objective functions have been proposed (Seireg and Arvikar, 1975; Crowninshield and Brand, 1981; Koopman et al., 1995).

Sparrow (1983) and Sparrow and Newell (1998) proposed a role for energy economy and described a novel perspective on how the motor control hierarchy is influenced. Their 1998 paper partially formalized this general perspective by recognizing that organismic, environmental, and task dependent factors can act as constraints to determine the movement strategy environment available while the selection of specific control patterns was guided by a ‘pressure to operate economically.’ Here we more rigorously define these factors.

It is relatively straightforward to conceptualize the potential influences of ‘organism’ and ‘environment.’ Organismic constraints encompass structural features such as proportions, mass distribution, allowable joint motions, etc., as well as physiological characteristics such as force and endurance limits, activation and contraction rates, and effective joint lever advantages. Environmental constraints include surface slope, coefficient of friction and intervening obstacles. The effects of both organism and environment are the motivation of substantial past and ongoing research (e.g., Taga, 1994; Manchester et al., 2011; Schoonaert et al., 2016).

In order to properly formalize the Sparrow and Newell construct, it is necessary to add to the concepts or organism and environment a clear definition of the task goal of locomotion and the process involved with the pressure to operate economically. In a previous article (Croft et al., 2017) we argue that the definition of the task in locomotion must be independent of the mechanisms/strategies that accomplish it, so that the problem(s) overcome with successful locomotion and the solutions utilized can be clearly distinguished. This allows the CNS selected solution to be evaluated in the context of the factors influencing the choices and the solution set of options properly mapped (in terms of both those selected and those avoided).

Contrary to common practice of defining the task of locomotion as the movements observed in successful locomotion, we suggest the task should be defined as the fundamental interaction of the organism with its external environment – which we see as the functionally optimal dynamic trajectory of the individual’s mass as it interacts with a supporting substrate. This leaves the commonly recognized features of locomotion task – limb kinematics, internal and external forces and their origin in neuromuscular activity – as the *mechanisms* that accomplish the task (Schroeder and Bertram, 2018). These mechanisms control the interaction of the mass with the substrate through the function of the legs, but it is the role of the CNS to employ those mechanisms to accomplish the (now explicitly defined) fundamental task. As such, these mechanisms are put into a functional context allowing interpretation of the value of strategies available to accomplish the task (or not – where considering unsuccessful strategies can be insightful in understanding why the preferred strategy is chosen). Putting movement strategies in a functional context allows an evaluation of the weighting of options, limitations, opportunities, selection of differing compromises, and a quantification of such important control characteristics as error assessment.

Having satisfied ourselves that definitions of the main constraints identified by Sparrow and Newell were possible, albeit dependent on a substantially different perspective of the task of locomotion (Croft et al., 2017), we sought a formalization of the suggested ‘pressure to operate economically.’ Based on the apparent influence of energetic cost derived from the studies discussed, we suggest that an assessment of energetic cost and the selection of less costly available options acts as the ‘pressure’ that guides the selection of movement strategy. This may occur in a broad range of motions but is particularly obvious in the energetically demanding context of locomotion.

It should be recognized that we are discussing motor control coordination at a different level than is usually the focus of gait coordination discussions. A common and fully legitimate question addressed in gait research asks, ‘how’ is locomotion coordinated? For that a substantial amount of rigorous study is used to produce insightful models directed at identifying the mechanisms that implement the strategy such as motor primitives (Dominici et al., 2011), synergies (Ivanenko et al., 2004; Cappellini et al., 2006), or planar covariation of kinematic control (Borghese et al., 1996; Ivanenko et al., 2004). Such models have been successful in describing how kinematics change early in the process of walking. As toddlers take their first few steps the elevation angles of the lower limb segments are quite variable, but within a few weeks the angles covary in a similar way to adults (Cheron et al., 2001). It is suggested that this development may occur to minimize energy expenditure. Hubel and Usherwood (2015) argue that toddlers’ gait is appropriate for their organismic constraints and is limited by muscle activation demands due to power, another optimization challenge, rather than energetic cost of transport *per se*. It is likely that very early in learning locomotion a basic neurologically determined movement pattern is utilized that is then modified through experience and feedback assessment to satisfy the requirements of dynamic stability, goal achievement (speed and direction) and to approach an energy-based optimization, with other factors such as not surpassing joint and muscle loading limits, avoiding obstacles, etc. also affecting the final strategy (which appears to rely on implementing leg kinematics that display planar covariation, Cheron et al., 2001).

In this review we have assumed that mechanisms exist to implement the strategy but ask the equally legitimate question, ‘why’ is a particular gait strategy implemented? In this we may well include the fundamental question of why normal locomotion is normal, as well as why specific parameter adjustments are made by the motor control system under specific circumstances (whether normal or not). We approach the problem following Srinivasan and Ruina (2006) and expect that almost any movement strategy is possible, then work to identify the factor that explains the broadest range of coordination adjustments – in order to gain insight into the limits and priorities of the coordination system. The location on the energetic cost surface that an individual operates at is substantially influenced by operational strategy of the limb as it manages the interaction of the mass of the individual with the substrate on which the individual moves – an interaction external to the body, so the emphasis of this review is on those interactions that occur at the external level. A comprehensive understanding of the motor control strategy and its influences must extend to interactions of the system (the individual) with the external environment – the purpose of the motions – rather than just to the cost involved with implementing the operational mechanisms within the body components.

The metabolic cost surface is a more rigorous definition of the energetic consequences of locomotion strategy than alluded to by Sparrow and Newell (1998). This surface defines the energetic advantages and consequences of any movement strategy selected. Although this surface is well documented for level locomotion on the treadmill, it is certain that the surface itself is susceptible to the effects of environmental circumstances (walking over obstacles, for instance) or features of the individual’s form and physiology (limb proportions, level of fatigue, proportion of muscle type, etc.). These influences, of course, match Sparrow and Newell’s environmental and organismic ‘constraints.’

**FIGURE 7:**
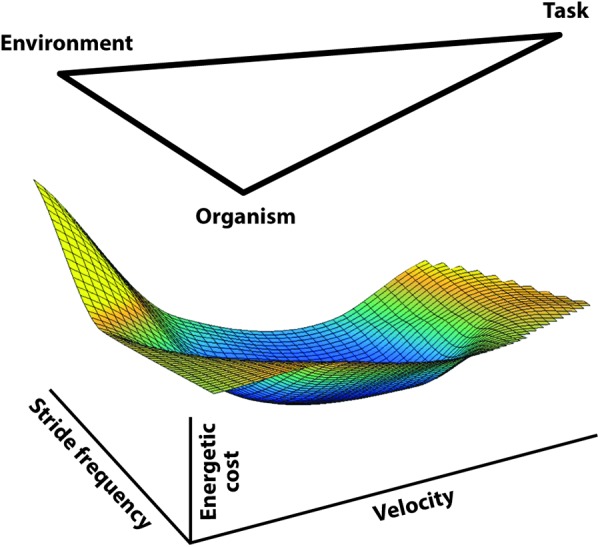
A diagram extending the concept of Sparrow and Newell (1998) economic movement constraints (environment, organism, and task) with the cost surface for walking implied as the outcome. Based on emerging evidence indicating that energetic cost has influence on the CNS control regime (reviewed herein), we propose that these ‘influences’ combine to determine the cost surface-motor control interaction. Energetically appropriate control strategies are generated by standard physiological feedback but as experience and learning accumulate a feed-forward anticipation of a serviceable control strategy develops. The feed-forward accommodation appears to extend to quite complex circumstances but likely is continually modified depending on the precise circumstances encountered.

Rather than constraints, we might suggest that these factors, including aspects of the task itself (such as speed of progression), can be considered influences on the location on the cost surface that the individual operates. Given a specific locomotory task, it is important for the CNS to determine the most appropriate action to implement. Because locomotion operates on an energetic cost surface, any movement strategy implemented will determine the location on that surface. The studies discussed above focused on the apparent sensitivity of the CNS to energetic cost and lead to a re-conceptualization of Sparrow and Newell (1998) constraint diagram, now including the energetic cost as an outcome (Figure 7). This conceptualization provides the basis for a more comprehensive perspective on the interaction of energetic cost and locomotion motor control, including the potential relationship between feedback and feed-forward effects.

Finally, why should energy be so important? In asymmetric split-belt walking, for instance, the individual is able to function without using an energy-minimizing modified motor control strategy, but individuals appear to universally adapt to a strategy that has an energetic advantage, even if that advantage is slight. Why should the motor control system be so sensitive to conserving relatively small amounts of energy?

Energy has some unique characteristics. As pointed out by Long and Srinivasan (2013), energy, like money, is a fungible resource – energy saved in one behavior is available to be used in another. Energy can be considered the currency of the body’s actions. It appears the motor control system is a prudent spender under most circumstances involving locomotion. Possibly this is because locomotion is such an important aspect of daily life (especially over evolutionary time). Even a subtle energy saving per step—over the course of days, months, or years—can sum to a valuable increase in a critical resource. To fully understand how, and why, the motor control system responds as it does, it will be necessary to investigate how the energy accounting books are kept. Again, this is an area of gait control that has received modest attention in the past, as compared to the detailed focus that has been given to mechanisms responsible for how control signals are generated, transferred, integrated, and expressed. We contend, however, that a complete understanding of motor coordination in gait must also consider the external aspects of the activity, amounting to the ‘why’ of locomotion. Doing so may well provide an important context for interpreting why the internal activities respond to energetic opportunities and consequences as they appear to.

## Author Contributions

JC and JB conceived the manuscript. JC, RS, and JB wrote and edited the manuscript.

## Conflict of Interest Statement

The authors declare that the research was conducted in the absence of any commercial or financial relationships that could be construed as a potential conflict of interest.

## References

[B1] Alexander R. M. (1991). Energy-saving mechanisms in walking and running. *J. Exp. Biol.* 160 55–69. 1960518 10.1242/jeb.160.1.55

[B2] Alexander R. M. N. (2002). Energetics and optimization of human walking and running: the 2000 Raymond Pearl memorial lecture. *Am. J. Hum. Biol.* 14 641–648. 10.1002/ajhb.10067 12203818

[B3] Bertram J. E. A. (2005). Constrained optimization in human walking: cost minimization and gait plasticity. *J. Exp. Biol.* 208 979–991. 10.1242/jeb.01498 15767300

[B4] Bertram J. E. A. (2015). Locomotion: why we walk the way we walk. *Curr. Biol.* 25 R795–R797. 10.1016/j.cub.2015.08.035 26394100

[B5] Bertram J. E. A. Gutmann A. Randev J. Hulliger M. (2014). Domestic cat walking parallels human constrained optimization: optimization strategies and the comparison of normal and sensory deficient individuals. *Hum. Mov. Sci.* 36 154–166. 10.1016/j.humov.2014.05.008 24974156

[B6] Bertram J. E. A. Hasaneini S. J. (2013). Neglected losses and key costs: tracking the energetics of walking and running. *J. Exp. Biol.* 216 933–938. 10.1242/jeb.078543 23447662

[B7] Bertram J. E. A. Ruina A. (2001). Multiple walking speed–frequency relations are predicted by constrained optimization. *J. Theor. Biol.* 209 445–453. 10.1006/jtbi.2001.2279 11319893

[B8] Borghese N. A. Bianchi L. Lacquaniti F. (1996). Kinematic determinants of human locomotion. *J. Physiol.* 494(Pt 3), 863–879. 10.1113/jphysiol.1996.sp021539 8865081 PMC1160684

[B9] Cappellini G. Ivanenko Y. P. Poppele R. E. Lacquaniti F. (2006). Motor patterns in human walking and running. *J. Neurophysiol.* 95 3426–3437. 10.1152/jn.00081.2006 16554517

[B10] Cheron G. Bengoetxea A. Bouillot E. Lacquaniti F. Dan B. (2001). Early emergence of temporal co-ordination of lower limb segments elevation angles in human locomotion. *Neurosci. Lett.* 308 123–127. 10.1016/S0304-3940(01)01925-5 11457575

[B11] Croft J. L. Schroeder R. T. Bertram J. E. A. (2017). The goal of locomotion: separating the fundamental task from the mechanisms that accomplish it. *Psychon. Bull. Rev.* 24 1675–1685. 10.3758/s13423-016-1222-3 28092079

[B12] Crowninshield R. D. Brand R. A. (1981). A physiologically based criterion of muscle force prediction in locomotion. *J. Biomech.* 14 793–801. 10.1016/0021-9290(81)90035-X 7334039

[B13] Dominici N. Ivanenko Y. P. Cappellini G. d’Avella A. Mondì V. Cicchese M. (2011). Locomotor primitives in newborn babies and their development. *Science* 334 997–999. 10.1126/science.1210617 22096202

[B14] Donelan J. M. Kram R. (1997). The effect of reduced gravity on the kinematics of human walking: a test of the dynamic similarity hypothesis for locomotion. *J. Exp. Biol.* 200 3193–3201. 9364025 10.1242/jeb.200.24.3193

[B15] Donelan J. M. Kram R. (2000). Exploring dynamic similarity in human running using simulated reduced gravity. *J. Exp. Biol.* 203 2405–2415. 10903155 10.1242/jeb.203.16.2405

[B16] Donelan J. M. Kram R. Kuo A. D. (2002). Simultaneous positive and negative external mechanical work in human walking. *J. Biomech.* 35 117–124. 10.1016/S0021-9290(01)00169-5 11747890

[B17] Farley C. T. McMahon T. A. (1992). Energetics of walking and running: insights from simulated reduced-gravity experiments. *J. Appl. Physiol.* 73 2709–2712. 10.1152/jappl.1992.73.6.2709 1490989

[B18] Finley J. M. Bastian A. J. Gottschall J. S. (2013). Learning to be economical: the energy cost of walking tracks motor adaptation. *J. Physiol.* 591 1081–1095. 10.1113/jphysiol.2012.245506 23247109 PMC3591716

[B19] Fujiki S. Aoi S. Funato T. Tomita N. Senda K. Tsuchiya K. (2015). Adaptation mechanism of interlimb coordination in human split-belt treadmill walking through learning of foot contact timing: a robotics study. *J. R. Soc. Interface* 12:0542. 10.1098/rsif.2015.0542 26289658 PMC4614464

[B20] Griffin T. M. Tolani N. A. Kram R. (1999). Walking in simulated reduced gravity: mechanical energy fluctuations and exchange. *J. Appl. Physiol.* 86 383–390. 10.1152/jappl.1999.86.1.383 9887153

[B21] Gutmann A. K. Bertram J. E. A. (2013). Constrained optimization of metabolic cost in human hopping. *Exp. Physiol.* 98 1178–1189. 10.1113/expphysiol.2012.069880 23538463

[B22] Gutmann A. K. Jacobi B. Butcher M. T. Bertram J. E. A. (2006). Constrained optimization in human running. *J. Exp. Biol.* 209 622–632. 10.1242/jeb.02010 16449557

[B23] Hasaneini S. J. Macnab C. J. B. Bertram J. E. A. Leung H. (2013). The dynamic optimization approach to locomotion dynamics: human-like gaits from a minimally-constrained biped model. *Adv. Robot.* 27 845–859. 10.1080/01691864.2013.791656

[B24] Hogan N. (1984). An organizing principle for a class of voluntary movements. *J. Neurosci.* 4 2745–2754. 10.1523/JNEUROSCI.04-11-02745.1984 6502203 PMC6564718

[B25] Hoyt D. F. Taylor C. R. (1981). Gait and energetics of locomotion in horses. *Nature* 292 239–240. 10.1038/292239a0

[B26] Huang H. J. Kram R. Ahmed A. A. (2012). Reduction of metabolic cost during motor learning of arm reaching dynamics. *J. Neurosci.* 32 2182–2190. 10.1523/JNEUROSCI.4003-11.2012 22323730 PMC3865509

[B27] Hubel T. Y. Usherwood J. R. (2015). Children and adults minimise activated muscle volume by selecting gait parameters that balance gross mechanical power and work demands. *J. Exp. Biol.* 218(Pt 18), 2830–2839. 10.1242/jeb.122135 26400978 PMC4582168

[B28] Ivanenko Y. P. Poppele R. E. Lacquaniti F. (2004). Five basic muscle activation patterns account for muscle activity during human locomotion. *J. Physiol.* 556(Pt 1), 267–282. 10.1113/jphysiol.2003.057174 14724214 PMC1664897

[B29] Jensen L. Prokop T. Dietz V. (1998). Adaptational effects during human split-belt walking: influence of afferent input. *Exp. Brain Res.* 118 126–130. 10.1007/s002210050262 9547070

[B30] Kistemaker D. A. Wong J. D. Gribble P. L. (2010). The central nervous system does not minimize energy cost in arm movements. *J. Neurophysiol.* 104 2985–2994. 10.1152/jn.00483.2010 20884757

[B31] Kistemaker D. A. Wong J. D. Gribble P. L. (2014). The cost of moving optimally: kinematic path selection. *J. Neurophysiol.* 112 1815–1824. 10.1152/jn.00291.2014 24944215 PMC4200004

[B32] Koopman B. Grootenboer H. J. de Jongh H. J. (1995). An inverse dynamics model for the analysis, reconstruction and prediction of bipedal walking. *J. Biomech.* 28 1369–1376. 10.1016/0021-9290(94)00185-7 8522549

[B33] Kram R. Domingo A. Ferris D. P. (1997). Effect of reduced gravity on the preferred walk-run transition speed. *J. Exp. Biol.* 200 821–826. 9076966 10.1242/jeb.200.4.821

[B34] Kuo A. D. (2001a). A simple model of bipedal walking predicts the preferred speed-step length relationship. *J. Biomech. Eng.* 123 264–269. 11476370 10.1115/1.1372322

[B35] Kuo A. D. (2001b). Energetics of actively powered locomotion using the simplest walking model. *J. Biomech. Eng.* 124 113–120. 11871597 10.1115/1.1427703

[B36] Kuo A. D. Donelan J. M. Ruina A. (2005). Energetic consequences of walking like an inverted pendulum: step-to-step transitions. *Exerc. Sport Sci. Rev.* 33 88–97. 10.1097/00003677-200504000-00006 15821430

[B37] Latash M. L. Turvey M. T. Bernstein N. A. (1996). *Dexterity and its Development.* London: Psychology Press.

[B38] Long L. L. Srinivasan M. (2013). Walking, running, and resting under time, distance, and average speed constraints: optimality of walk-run-rest mixtures. *J. R. Soc. Interface* 10:20120980. 10.1098/rsif.2012.0980 23365192 PMC3627106

[B39] Malone L. A. Bastian A. J. Torres-Oviedo G. (2012). How does the motor system correct for errors in time and space during locomotor adaptation? *J. Neurophysiol.* 108 672–683. 10.1152/jn.00391.2011 22514294 PMC4073916

[B40] Manchester I. R. Mettin U. Iida F. Tedrake R. (2011). Stable dynamic walking over uneven terrain. *Int. J. Rob. Res.* 30 265–279. 10.1177/0278364910395339 27423264

[B41] Mawase F. Haizler T. Bar-Haim S. Karniel A. (2013). Kinetic adaptation during locomotion on a split-belt treadmill. *J. Neurophysiol.* 109 2216–2227. 10.1152/jn.00938.2012 23365187

[B42] Nessler J. A. Gilliland S. J. (2009). Interpersonal synchronization during side by side treadmill walking is influenced by leg length differential and altered sensory feedback. *Hum. Mov. Sci.* 28 772–785. 10.1016/j.humov.2009.04.007 19796834

[B43] Polet D. T. Schroeder R. T. Bertram J. E. A. (2018). Reducing gravity takes the bounce out of running. *J. Exp. Biol.* 221:162024. 10.1242/jeb.162024 29217625

[B44] Prokop T. Berger W. Zijlstra W. Dietz V. (1995). Adaptational and learning processes during human split-belt locomotion: interaction between central mechanisms and afferent input. *Exp. Brain Res.* 106 449–456. 10.1007/BF00231067 8983988

[B45] Reisman D. S. Block H. J. Bastian A. J. (2005). Interlimb coordination during locomotion: what can be adapted and stored? *J. Neurophysiol.* 94 2403–2415. 10.1152/jn.00089.2005 15958603

[B46] Richardson M. J. Campbell W. L. Schmidt R. C. (2009). Movement interference during action observation as emergent coordination. *Neurosci. Lett.* 449 117–122. 10.1016/j.neulet.2008.10.092 18996439

[B47] Sawicki G. S. Ferris D. P. (2009). Powered ankle exoskeletons reveal the metabolic cost of plantar flexor mechanical work during walking with longer steps at constant step frequency. *J. Exp. Biol.* 212 21–31. 10.1242/jeb.017269 19088207

[B48] Schoonaert K. D’Août K. Samuel D. Talloen W. Nauwelaerts S. Kivell T. L. (2016). Gait characteristics and spatio-temporal variables of climbing in bonobos (*Pan paniscus*). *Am. J. Primatol.* 78 1165–1177. 10.1002/ajp.22571 27309794

[B49] Schroeder R. T. Bertram J. E. A. (2018). Minimally actuated walking: identifying core challenges to economical legged locomotion reveals novel solutions. *Front. Robot. AI* 5:58 10.3389/frobt.2018.00058 PMC7904315 33644120

[B50] Seireg A. Arvikar R. J. (1975). The prediction of muscular load sharing and joint forces in the lower extremities during walking. *J. Biomech.* 8 89–102. 10.1016/0021-9290(75)90089-5 1150683

[B51] Selgrade B. P. Toney M. E. Chang Y.-H. (2017). Two biomechanical strategies for locomotor adaptation to split-belt treadmill walking in subjects with and without transtibial amputation. *J. Biomech.* 53 136–143. 10.1016/j.jbiomech.2017.01.012 28126335 PMC5340589

[B52] Selinger J. C. O’Connor S. M. Wong J. D. Donelan J. M. (2015). Humans can continuously optimize energetic cost during walking. *Curr. Biol.* 25 2452–2456. 10.1016/j.cub.2015.08.016 26365256

[B53] Snaterse M. Ton R. Kuo A. D. Donelan J. M. (2011). Distinct fast and slow processes contribute to the selection of preferred step frequency during human walking. *J. Appl. Physiol.* 110 1682–1690. 10.1152/japplphysiol.00536.2010 21393467 PMC4182286

[B54] Sparrow W. A. (1983). The efficiency of skilled performance. *J. Mot. Behav.* 15 237–261. 10.1080/00222895.1983.10735299 15151872

[B55] Sparrow W. A. Newell K. M. (1998). Metabolic energy expenditure and the regulation of movement economy. *Psychon. Bull. Rev.* 5 173–196. 10.3758/BF03212943

[B56] Srinivasan M. Ruina A. (2006). Computer optimization of a minimal biped model discovers walking and running. *Nature* 439 72–75. 10.1038/nature04113 16155564

[B57] Taga G. (1994). Emergence of bipedal locomotion through entrainment among the neuro-musculo-skeletal system and the environment. *Physica D* 75 190–208. 10.1016/0167-2789(94)90283-6

[B58] Wong J. D. Donelan J. M. (2017). “Principles of energetics and stability in legged locomotion,” in *Humanoid Robotics: A Reference*, eds Goswami A. Vadakkepat P. (Dordrecht: Springer), 1–28.

